# Virtual reality aggression prevention treatment in a Dutch prison-based population: a pilot study

**DOI:** 10.3389/fpsyg.2023.1235808

**Published:** 2023-11-14

**Authors:** Kasja Woicik, Chris N. W. Geraets, Stéphanie Klein Tuente, Erik Masthoff, Wim Veling

**Affiliations:** ^1^Penitentiary Institution Vught, Vught, Netherlands; ^2^Department of Psychiatry, University of Groningen, University Medical Center Groningen, Groningen, Netherlands; ^3^Lund Clinical Research on Externalizing and Developmental Psychopathology, Child and Adolescent Psychiatry, Department of Clinical Sciences Lund, Lund University, Lund, Sweden; ^4^Fivoor Science and Treatment Innovation, Rotterdam, Netherlands; ^5^Department of Developmental Psychology, Tilburg University, Tilburg, Netherlands

**Keywords:** aggression, virtual reality, prison, behavioral training, forensic

## Abstract

**Background:**

Treating violent behavior in prisons comes with challenges, such as the inability to practice safely with triggering situations and motivational issues. A solution may be the use of Virtual Reality (VR). With VR, specific conditions or needs can be tailored for individual practice, it can enhance motivation and VR has proven to be a safe and effective tool in mental health treatment.

**Objective:**

A pilot study was conducted to test the acceptability, feasibility, and preliminary effects of VR Aggression Prevention Treatment (VRAPT) in a prison-based population.

**Methods:**

In total 17 detainees with aggressive behavior were included in this single-group pilot study. Acceptability and feasibility were assessed using qualitative measures for participants and therapists. Preliminary treatment effects were measured with self-report and observational measures on aggression, anger, emotion regulation, and impulsiveness.

**Results:**

Participants and therapists were predominantly positive about VRAPT. Participants rated the sessions with an average satisfaction score of 9.2 out of 10 (SD = 0.3). Qualitative data showed that participants reported having learned to respond more adequately to aggressive behavior and gained insights into their own and others’ triggers and tension. The combination of VR and theory was experienced as a strength of the treatment, as well as the ability to trigger aggression in VR which provided insights into aggression. However, the theoretical framework was found to be too complex, and more aggressive and personal scenarios should be incorporated into the sessions. Self-reported aggression, anger, provocation, emotion regulation, and observed verbal aggression decreased and seemed to stabilize after the treatment ended, with small to medium effect sizes.

**Conclusion:**

VRAPT proved feasible and acceptable for most participants and therapists. An adapted treatment protocol called Virtual Reality Treatment for Aggression Control (VR-TrAC), will be used in a future RCT to investigate the effects of the treatment in a prison-based population.

## 1 Introduction

Offenders of violent crimes involving force or causing injury (such as assault, homicide or armed robbery) recidivate more often than non-violent offenders (Cuervo et al., 2018; Recidivism Among Federal Violent Offenders, 2019). Furthermore, in prison violent offenders show high rates of aggression and violence, causing harm to staff, fellow detainees, and their living environment (Mcguire, 2018; Mcneeley, 2020; Wildra, 2020). Aggressive behavior during imprisonment has also been related to higher rates of crime recidivism (Mooney and Daffern, 2015). Given these effects of aggression on both society and the prison environment, treating individuals with aggression problems during detention is of critical need. Whereas social interventions such as supporting work and education have been found to be related to reduced recidivism, also psychological treatment forms are an important strategy for decreasing recidivism and violent behavior (Lipsey and Cullen, 2007; Walk et al., 2021).

Research has shown that, in general, psychological aggression treatment can be successful in reducing such behavior with small to moderate effect sizes (McGuire, 2008). However, findings for prison-based populations are still inconclusive. A recent meta-analytic review concluded that more high-quality research is needed to understand the specific factors contributing to effective treatment (Papalia et al., 2019). Research on prisoners has shown that, in general, psychological therapies that combine more than one method, seem most effective (Auty et al., 2017; Papalia et al., 2019). Such treatments mostly consist of multimodal cognitive behavioral methods that focus on role-play, relapse prevention, reshaping cognitions, improving problem-solving, exposure, and/or training skills (Papalia et al., 2019).

One of the best-known and studied frameworks for treating prison-based populations is the Risk-Need -Responsivity model (RNR). This model states that interventions should be personalized based on the risk of recidivism (Risk), adjusted to the factors that predict criminogenic behavior (behavior directly related to recidivism; Need) and should fit the motivation and abilities of the offender (Responsivity; Polaschek, 2012). A common limitation concerning this “Need” principle in treating violent behavior in prison-based populations is the inability to practice safely with challenging and triggering real-life situations. Furthermore, motivational issues are common in such populations, for example, because individuals may lack problem awareness or may have followed therapy previously without success, demotivating them to follow therapy again (Jochems et al., 2012; Smeijers et al., 2018). Also, interventions often have a cognitive and theoretical approach, which may not be the best fit for prison-based populations in which intellectual abilities below average are common, limiting responsivity (Muñoz García-Largo et al., 2020).

A solution to the above-encountered problems may be the use of Virtual Reality (VR; Kip et al., 2018, 2019). VR uses computer-generated, interactive environments to imitate real-world situations. VR makes it possible to practice situations in a virtual environment and therefore individuals can practice aggression-inducing interactions safely. Virtual situations can also be tailored to fit the specific conditions or needs of an individual to practice (Freeman et al., 2017; Kip et al., 2019). Furthermore, adding VR technology as a tool in treatment may enhance motivation, as it is new, interesting, and interactive technology. In general, VR has been proven to be a safe and effective tool in the treatment of several disorders, such as anxiety and psychotic disorders (Freeman et al., 2017; Geraets et al., 2021). However, as a recent review on VR in forensic settings discussed, studies using VR in the treatment of behavior (such as aggression) show promising results but the number of studies is still very limited and further research is needed (Sygel and Wallinius, 2021).

A recent multicenter randomized controlled trial (RCT) by Klein Tuente et al. investigated the first VR aggression prevention training (VRAPT) in forensic inpatients (Klein Tuente et al., 2020). As a theoretic underpinning the social information processing theory (SIP) was used which is based on the social-cognitive theory (Dodge and Crick, 2007; Klein Tuente et al., 2018). To understand aggressive behavior, the SIP describes several steps in which an individual processes social and situational information, based on which a behavioral response is enacted (Crick and Dodge, 1994). Within the framework of the SIP model, aggressive behavior can result from aberrant or biased interpretation of situations, but also from aggression-inducing goal framing, a learning history impacted by trauma or aggressive role models, and limited resources to respond adequately (for more details of the SIP model see the methods section).

The participants were long-term forensic inpatients (average duration since index offense was 8 years) with a special judicial measure, called ‘TBS-order’ which is a measure for the court establishing a relationship between the committed crime and a psychiatric disorder. The forensic inpatients were positive about VRAPT and motivated to participate in the RCT, which was reflected by high inclusion rates (Klein Tuente et al., 2020). Furthermore, interviews revealed that participants were able to recall what they had learned (e.g., recognizing arousal and insights in triggers). Whereas no decrease in staff-rated or self-reported aggression was found in this RCT, self-reported hostility, anger and impulsiveness did improve after VRAPT compared to the waiting list condition. The lack of effects on aggression might be explained by the study population as it concerned participants with severe and long-lasting mental illness, who had been treated for many years. Given the long-term treatment history and persistent psychiatric problems, the included population may have had limited abilities to change.

In the current study, we aimed to test the VRAPT protocol in a prison-based population. To our knowledge, this is the first study examining VR treatment for aggression problems in a prison-based setting. Although no significant changes in aggression were found in the first VRAPT study, the intervention fits well within the RNR framework for prison-based populations. Though similar levels of motivation are expected, higher responsivity is expected as prisoners with aggression regulation problems likely have no, shorter-term and/or less severe psychopathology than long-stay forensic inpatients. Therefore, with this pilot, we aimed to test the acceptability, applicability and feasibility of VRAPT and identify potential points of improvement in the treatment protocol, as a base for a future RCT. The secondary objective was to explore the preliminary effects of VRAPT on aggression, anger, emotion regulation and impulsive behavior directly after treatment and at two-month follow-up.

## 2 Methods

### 2.1 Participants

Participants were male detainees residing at the Penitentiary Institution Vught in the Netherlands. On the units where participants were recruited, only male detainees were imprisoned. The study was announced through flyers and a video on the prison tv-channel, and detainees were made aware of the study when aggressive behavior was noticed in them by the staff. Detainees who wanted to participate applied themselves by asking the staff for contact with the researchers.

Inclusion criteria were aged 18 or older and aggression regulation problems within the last month as indicated with the Aggression Questionnaire (AQ), with a score > 70 (Buss and Perry, 1992). Exclusion criteria were an indication of an intellectual disability (measured with the Screener for intelligence and mild intellectual disability (SCIL) score < 15 which is indicative of an IQ below 70; van Esch et al., 2020), acute suicidality, current psychotic episode, occurrence of epileptic seizures within the past year, insufficient command and understanding of the Dutch language, and estimated remaining imprisonment shorter than 5 months.

We aimed to include approximately 15 participants. The final sample consisted of 17 participants of whom the average age was 32 years (SD 8.4). Participants were of different ethnicity, foremost being Dutch (59%), but also Moroccan (12%), Colombian (6%), Antillean (6%), Congolese (6%), Belgian (6%), and Surinamese (6%). Participants had different educational levels: five participants (31%) had none or a lower education level, four (25%) had a vocational educational level, five (31%) had a secondary vocational level and two (12%) had a higher educational level. The majority of participants were single and had children (44%), 38% were single and did not have children, 17% had children and lived with a partner. Further characteristics of the sample are shown in Table 1.

**Table 1:** Baseline characteristics of the sample (*N* = 17).

	M(SD) or *N* (%)	
	Completers *N* = 10	Drop-out *N* = 7
Age	32.2 (8.2)	32.4 (9.4)
Education level		
None or primary	4 (40%)	1 (14.3%)
Vocational	3 (30%)	1 (14.3%)
Secondary vocational	1 (10%)	4 (57.1%)
Higher	1 (10%)	1 (14.3%)
Convictions		
Manslaughter	3 (30%)	1 (14.3%)
Property crimes (with violence)	2 (20%)	4 (57.1%)
(Heavy) violent crimes	2 (20%)	1 (14.3%)
Homicide	2 (20%)	0
Property crime (without violence)	1 (10%)	1 (14.3%)
Arson	1 (10%)	0
Destruction (property)	1 (10%)	0
Traffic violation	1 (10%)	0
Adverse childhood experiences		
Emotional abuse	1 (10%)	4 (57.1%)
Physical abuse	2 (20%))	4 (57.1%)
Sexual abuse	3 (30%)	1 (14.3%)
Emotional neglect	2 (20%)	4 (57.1%)
Physical neglect	0	2 (28.6%)
Parental separation or divorce	4 (40%)	5 (71.4%)
Mother treated violently	1 (10%)	2 (28.6%)
Household substance abuse	0	4 (57.1%)
Mental illness in household	0	2 (28.6%)
Criminal household member	3 (30%)	3 (42.9%)
Substance abuse		
Alcohol	3 (30%)	1 (16.7%)
Tobacco	2 (20%)	1 (16.7%)
Cannabis	4 (40%)	2 (33.3%)
Cocaine	0	1 (16.7%)

### 2.2 Design and procedure

This was an uncontrolled pilot intervention study with three measurement moments. This study was approved by the Medical Ethical Committee of the University Medical Center Groningen (METC number: 2019/381). Participants were not compensated for participating.

When a detainee was interested in the study, a researcher visited the detainee and provided verbal and written information on the study. If the detainee was willing to participate, informed consent was signed. For the screening, the SCIL score was checked from their file and the AQ was administered (van Esch et al., 2020).

After informed consent was obtained, observations by the staff started and continued until 4 weeks after the last VRAPT session had taken place. Four weeks after the start of the observations, the baseline assessment was performed. Then the treatment took place and the post-treatment assessment was performed after the final session. In case of treatment dropout, the post-treatment assessment was performed 2 months after the baseline assessment. The follow-up assessment was performed 2 months after post-treatment. All participants received care as usual, when necessary.

### 2.3 VR system

**Figure 1:**
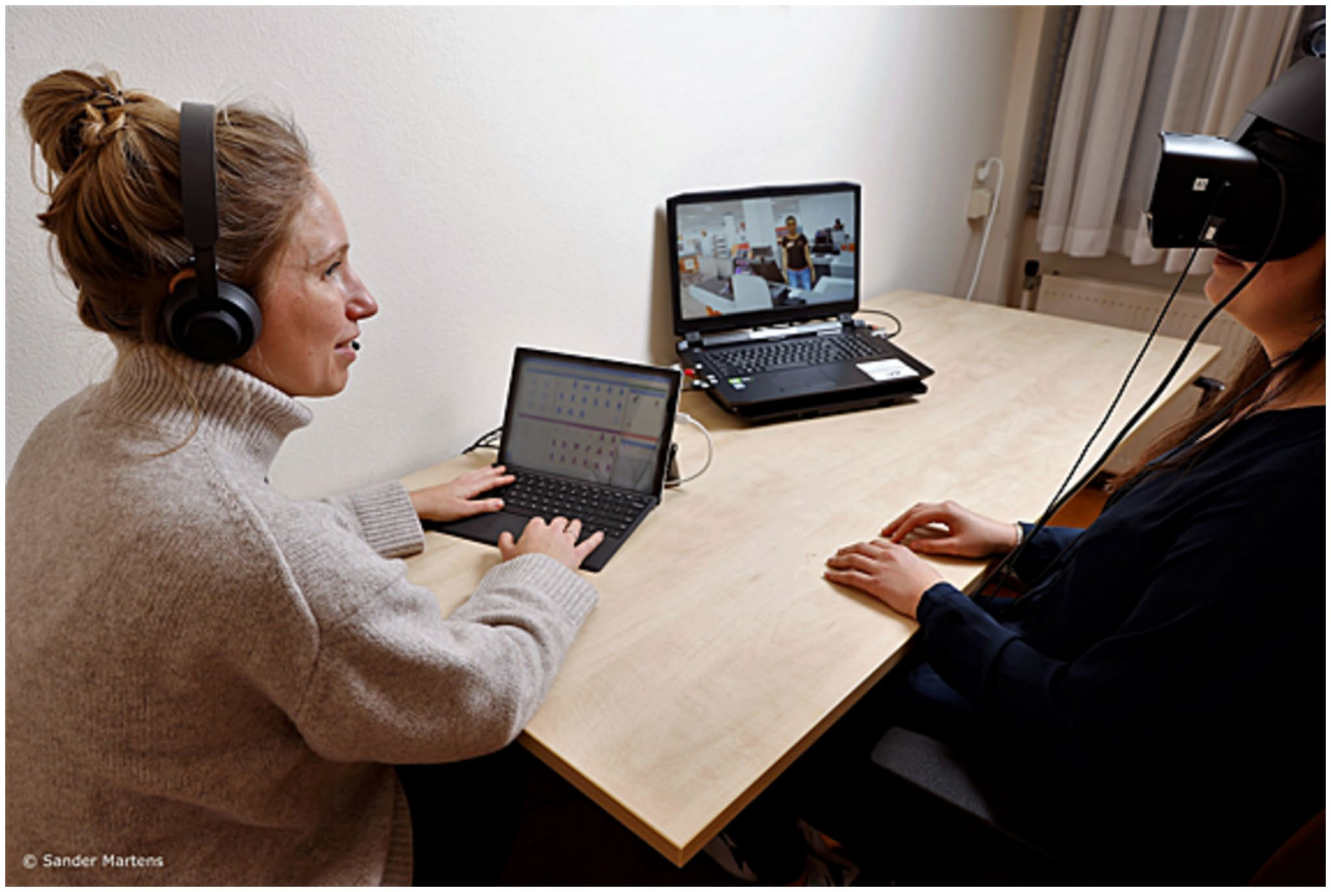
The VR set-up. Reproduced with permission from Sander Martens.

**Figure 2:**
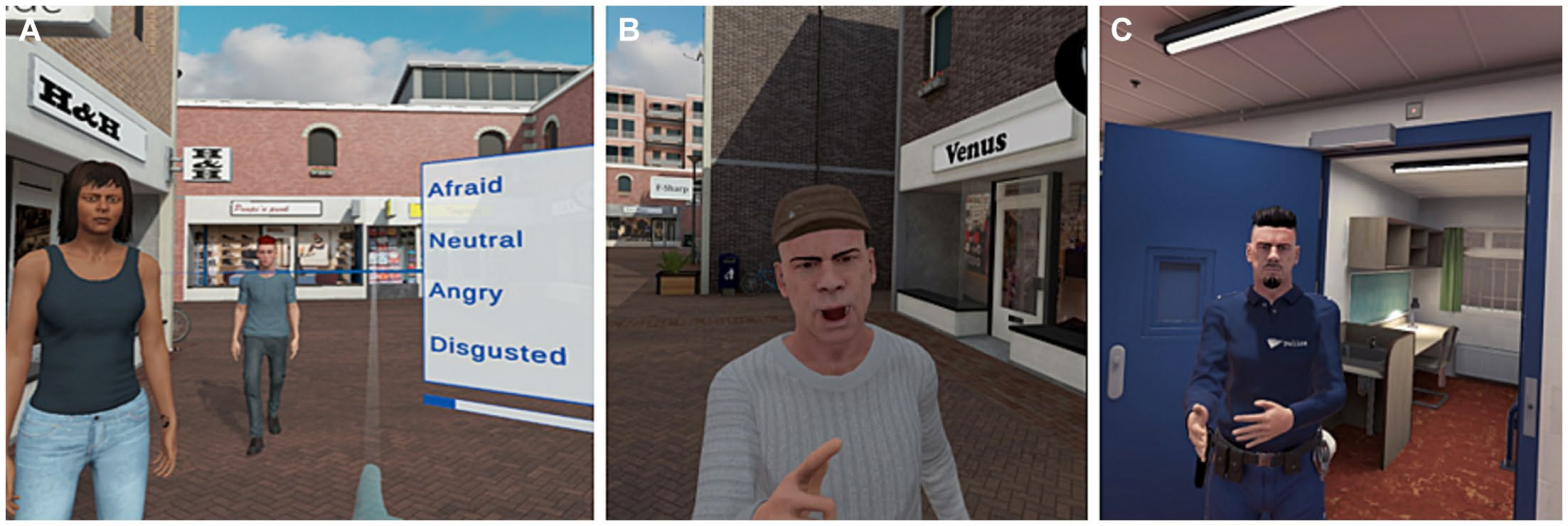
Impression of the **(A)** VR emotion recognition task, **(B)** aggression catwalk, and **(C)** an interactive scenario. Images of the VR environment are reproduced with permission from CleVR BV.

Participants were exposed to simulated virtual environments by wearing an Oculus Rift S headset and noise-canceling headphones, see Figure 1. Therapists operated the VR surroundings with a tablet, and on a second screen the therapist saw what the participant viewed. The ‘Social Worlds’ software, created with Unity by CleVR BV was used in this study, which was also used in the first VRAPT RCT (Klein Tuente et al., 2020). The following three modules of the software were used: (1) the emotion recognition task, (2) the aggression catwalk, and (3) the interactive scenarios. See Figure 2 for screenshots of the software.

During the emotion recognition task, participants navigated the virtual street by changing their body orientation and operating a joystick enabling forward and backward movement. Avatars were standing at random locations in the VR street. When a participant moved within a two-meter radius, the avatar oriented towards the participant and displayed an emotion (anger, disgust, fear, happiness, sadness, surprise, or neutral), and the correct emotion had to be chosen from a pop-up menu with four options with the joystick. Six pre-installed levels were available to enable customizing.

In the aggression catwalk, participants were approached by avatars showing neutral to aggressive behavior. This was shown through facial emotions, body language and verbal expressions. Participants rated the level of aggression (from level 1 not aggressive to level 4 very aggressive).

During interactive scenarios, therapists wore headphones with a microphone and voice morphing. The virtual environments (e.g., a store, bar or prison) could be adapted to the specific needs of the participants, for example by choosing specific avatars (e.g., a security guard, a group of females or males with different ethnic backgrounds) to be present in the VR environment, as well as the number and type of avatars in the background. Furthermore, the therapist controlled the emotions, gestures and speech of the avatar(s) with whom the participant was interacting.

### 2.4 Intervention

The treatment consisted of 16 twice-weekly individual sessions with a maximum duration of 60 min per session. In practice, participants complete VRAPT on average in 13 weeks (range 8–26 weeks), due to practical reasons such as sickness of the participant or therapist, COVID restrictions, no-show of the participants or vacation of the therapist. Sessions were planned in consultation with the participant. Four qualified psychologists received a one-day training and monthly group supervision.

The treatment protocol used the SIP model as a theoretical framework, which describes how problems with social information processing are linked to aggressive behavior (Crick and Dodge, 1994). It describes six steps in which an individual processes social and situational information leading to behavioral responses. The early stages involve the identification of (1) what is happening and (2) what it means to me. The late stages match the outcomes of (1) and (2) to (3) what goals am I trying to achieve, (4) what options do I have to react, (5) what am I going to do, eventually culminating in (6) the reaction or behavior. The steps are interrelated and can influence each other. During the treatment, each step of the SIP model is discussed, and related exercises are performed to improve social information processing and practice new behavior.

The treatment consisted of two parts. Part one focused on the early stages of social information processing, related to emotion recognition (sessions 2–6). Part two focused on the late information processing stages with interactive scenarios (sessions 7–15).

Each session started with a short recap of the previous session. During this recap, participants were asked if they discussed the theory with their mentor (every prisoner has an individual mentor) if they experienced any situations relevant to VRAPT, and if they applied any learned skills. Next, the theory and goal of the current session were explained and VR exercises were performed. The session ended with discussing how the participant could apply the learned theory and skills in the upcoming week. Participants received a workbook that contained exercises and a summary of the theory discussed in each session. From session 6 onwards, physiological measures were performed during the session. Below an overview of the sessions is given.

**Session 1:** included a general introduction and a simplified version of the SIP model was explained. At the end of the session, participants got acquainted with the VR system by exploring the VR street by utilizing the joystick, for maximally 10 min.

**Session 2:** SMART treatment goals were formulated and expectations were discussed. The first two steps of the SIP model and the relation between emotion recognition and emotions were discussed shortly as it is known that individuals with aggression problems are more likely to interpret the behavior of others as aggressive (especially when ambiguous or unpredictable; Coccaro et al., 2017). Then participants practiced with three levels of the emotion recognition task. This was customized based on the performance of the participant on the task.

**Sessions 3:** the information of session 2 was summarized. Then participants practiced the emotion recognition task again. The difficulty level could be adjusted to the skills of the participant.

**Session 4:** focused on recognizing different levels of aggression. After discussing this topic, participants practiced with the aggression catwalk in VR. Participants rated approximately 10 to 15 avatars on their level of aggression. Scores were evaluated and the participant repeated the task.

**Session 5:** learning goals were evaluated and the theory from sessions 1 to 4 was repeated. The therapist discussed with the participant where repetition was needed, and exercises from the previous session were repeated (i.e., the emotion recognition task or aggression catwalk).

**Sessions 6:** information on steps 3 to 6 of the SIP model was repeated (they were explained in session 1) and the concept of physical arousal and physiological measurements were introduced (heart rate measured with an electrocardiogram (ECG), and galvanic skin response (GSR) measured with a finger sensor). The goal was to use these measures to teach participants to recognize physical signs of arousal. Real-time graphs of the physiological measures, the VR, and the therapist’s and participant’s voices were recorded to enable rewatching and discussing physiological responses with the participant. While participants wore the sensors, they were approached aggressively by avatars during the aggression catwalk. Participants were asked not to react to the avatars but to pay attention to signals in their body. This was discussed after the assignment. Next, participants were approached again by aggressive avatars on the aggression catwalk, but now they had to react like they normally would. The experience was discussed.

**Session 7:** the goal was to learn that there are different responses to aggression-provoking situations. This was practiced with two pre-scripted interactive scenarios. Scenario 1 took place in the bar, where the participant is accused of louring at the girlfriend of an avatar. The participant was asked to react like he would normally do. After the scenario, different types of reactions were discussed (sub-assertive, assertive, and aggressive), and the participant’s reaction was classified. The assertive way was discussed in more detail (telling your message in an I-formulated message). Next, three interactive scenarios were played in which the participants spilled coffee on the shoes of the avatar in the bar. The therapist (enacting the avatar) demonstrated the different types of reactions in each scenario to give more insight into the different responses and what they provoke.

**Session 8:** different responses were practiced in three interactive scenarios in the supermarket. The participant wanted to enter the supermarket, but the security denied his entrance due to closing time. However, during the scenario the participant saw how another avatar entered the supermarket without the security noticing it. In the first scenario, it was asked to react in a sub-assertive way, in the second in an assertive way and the third in an aggressive way. The different ways of reacting were then discussed and evaluated.

**Session 9:** different ways and tools were discussed to help the participant react more assertively, including strategies such as ignoring, helping thoughts, counting to 10, focusing on breathing, or a time-out. In this session, three prescripted scenarios were played. In scenario 1 he was accused by a police officer of something he did not do, in scenario 2 the participant was not allowed to call his lawyer and in scenario 3 he had an argument with friends. Before each scenario, it was discussed which strategy the participant wanted to use. The scenarios were discussed and evaluated afterward.

**Session 10:** three different pre-scripted scenarios were played to practice new skills and reduce tension. In the first the participant was accused of stealing something, in the second scenario the participant wanted to order a drink but the bartender refuses and in the third scenario, the participant was accused of using drugs in prison. The participant practiced with the strategies that were discussed in session 9.

**Session 11:** goals were evaluated and a brief recap of the different kinds of responses and strategies was given. One or two interactive scenarios were practiced. The scenario could be chosen from a list of pre-scripted scenarios, or a personalized scenario could be practiced (with a personalized environment, number and type of avatars as well as general content of the interaction).

**Session 12–15:** Three personalized or pre-scripted scenarios per session were performed. Each scenario was discussed and evaluated.

**Session 16**: the treatment was evaluated, and when necessary, previous topics were repeated or trained with an interactive scenario.

### 2.5 Measures

#### 2.5.1 Sociodemographic and clinical characteristics

Sociodemographic characteristics were collected on age, cultural background, education, family status, and conviction history. Lifetime substance dependence and abuse were measured with the *Measurement in the Addiction for Triage & Evaluation* (*MATE*; Schippers et al., 2011). The MATE has good psychometric standards, with satisfactory inter-rater reliability (ICC range 0.75–0.92). Concurrent validity is good, with correlations above 0.50 (Schippers et al., 2010). Childhood trauma was measured with the *Adverse Childhood Experiences* (*ACE*; van der Feltz-Cornelis et al., 2019). Construct reliability is acceptable (ω = 0 0.91; Mei et al., 2022).

#### 2.5.2 Self-report measures

At baseline, post-treatment, and follow-up the following questionnaires were administered.

Aggression was measured with the *Aggression Questionnaire (AQ)* which was the primary outcome measure (Buss and Perry, 1992). The AQ consists of 29 items measuring aggression on four different scales: physical aggression, verbal aggression, anger, and hostility. Items are rated on a 5-point Likert scale (ranging from ‘disagree a lot’ to ‘agree a lot’). Test–retest reliability of the AQ is good (0.72), as well as the internal consistency (Cronbach’s α = 0.83). This also applies to the validity of the total score of the AQ (the AQ correlated positively with alternative questionnaires measuring aggression; Hornsveld et al., 2009).

Anger was assessed with the *Novaco Anger Scale and Provocation Inventory* (*NAS-PI*; Hornsveld et al., 2011), which consists of two parts. The NAS part contains 48 questions and measures three factors of anger: cognitive, arousal, and behavior. Items are measured on a 3-point Likert scale (ranging from ‘never true’ to ‘always true’). The PI part contains 25 items assessing provocation in response to anger-eliciting situations rated on a 4-point Likert scale (ranging from ‘not angry at all’ to ‘very angry’). The internal consistency of the NAS and PI is excellent (Cronbach’s α NAS = 0.92, and Cronbach’s α PI = 0.90, the test–retest reliability of the NAS is good (r = 0.80) and the validity of the NAS and PI is good (the NAS-PI correlated positively with alternative questionnaires measuring anger and personality; Hornsveld et al., 2011).

Reactive and proactive aggression was measured with the *Reactive-Proactive Questionnaire* (*RPQ*; Cima et al., 2013). The RPQ consists of 23 items rated on a 3-point Likert scale (ranging from ‘never’ to ‘a lot’); 11 items on reactive aggression and 12 items on proactive aggression. The RPQ has excellent internal consistency (Cronbach’s α = 0.91), test–retest reliability is good, (all ICCs>0.41 at 3-year follow-up) and the convergent validity is adequate (with significant positive correlations with several other aggression measures; Cima et al., 2013).

Emotion regulation was assessed with the *Difficulties in emotion regulation* (*DERS*; Gratz and Roemer, 2004). The DERS consists of 36 items measured on a 5-point Likert scale (ranging from ‘almost never’ to ‘almost always’). The DERS has high internal consistency (Cronbach’s α = 0.93 for the total score), good test–retest reliability (r = 0.88; Gratz and Roemer, 2004).

Impulsive behavior was measured with the *Baratt Impulsiveness Scale* (*BIS-11*; Stanford et al., 2009). The BIS-11 has 30 items, assessing different personality and behavioral constructs of impulsiveness, rated on a 4-point Likert scale (ranging from ‘rarely/never’ to ‘almost always’). The BIS-11 showed good internal consistency (Cronbach’s α = 0.83) and test–retest reliability (Spearman‘s Rho = 0.83; Stanford et al., 2009).

#### 2.5.3 Staff-rated measure

Aggressive behavior was scored by prison staff with the *Social Dysfunction and Aggression Scale* (*SDAS-9*; Wistedt et al., 1990; Kobes et al., 2012). The SDAS-9 is a behavior-observatory questionnaire consisting of 9 items measuring the extent of outward physical and verbally aggressive behavior in the past week. It is rated on a 5-point Likert scale, ranging from ‘not present’ to ‘very serious’. Internal consistency (Cronbach’s α = 0.82) and convergent validity are good (r = 0.73 with the staff observation aggression scale revised and interobserver reliability is moderate (ICC = 0.50; Kobes et al., 2012).

#### 2.5.4 VR session measures

At the end of each session, participants completed the *Session Rating Scale (SRS)* on session satisfaction (Duncan et al., 2003) which includes the therapeutic alliance. The SRS consists of four items measuring the relationship (from “I did not feel heard, understood, and respected” to “I did feel heard, understood, and respected”), goals and topics (from “We did not work on or talk about what I wanted to work on and talk about” to “We did work on or talk about what I wanted to work on and talk about”), approach or method (from “The therapist’s approach is not a good fit for me” to “The therapist’s approach is a good fit for me”) and the fourth item requires the participant to generally evaluate the session. Items are scored on a 10-centimeter visual analog scale, ranging from 0 to 10. The total score was analyzed. The SRS shows moderate to high internal consistency (ranging from 0.70 to 0.97) and low to moderate validity (ranging from 0.29 to 0.48; Murphy et al., 2020).

The sense of presence experience in VR was measured with *The Igroup Presence Questionnaire (IPQ)* and was conducted at the end of treatment (Schubert et al., 2001). The IPQ is rated on a 7-point Likert scale, ranging from negative statements about the VR world (such as ‘the VR world felt like an imaginary world) to positive statements about the VR world (such as ‘it could not be differentiated from the real world’). Internal consistency reliability is good (Cronbach’s α = 0.85; Igroup, n.d.).

#### 2.5.5 Qualitative measurements

Therapists and participants completed open questions about the treatment after every session. The questions concerned how they generally experienced the session, how well the session content fitted the participant’s aggression regulation problem, critical feedback about the session, if there were any difficulties, how they experienced the use of the VR equipment, the exercises and roleplays, and the usage of the treatment protocol. During the first session, additionally participants were specifically asked whether they experienced cybersickness, in the following sessions this was only noted when reported by the participant.

### 2.6 Analyses

Qualitative data consisted of answers to open questions that were first grouped into topics by the first author. Then, similar answers and categories were grouped in an iterative process between the first and second authors. After sessions 2 to 15, the therapists rated how well the session content fitted the participant’s aggression problem. Answers were coded into a good fit, reasonable fit, or did not fit.

For quantitative data, descriptive statistics were calculated, i.e., means and standard deviations or count and percentages. Total scores were calculated if maximally 2 items were missing, for subscales maximally 1 item was allowed to be missing. Missing items were replaced by the scale mean item score. Effect sizes were calculated based on the mean and standard deviation for parametric data and the ‘Hedges’ g correction was used because of the small sample size (n < 20; 0.20–0.49 is a small effect, 0.50–0.79 medium effect and 0.8 a large effect). For non-parametric data effect sizes were based on the Wilcoxon signed rank test for which effect sizes 0.10–0.39 are considered a small effect, 0.30–0.49 a medium effect and 0.50 or higher a large effect.

## 3 Results

### 3.1 Feasibility and acceptability

**Figure 3:**
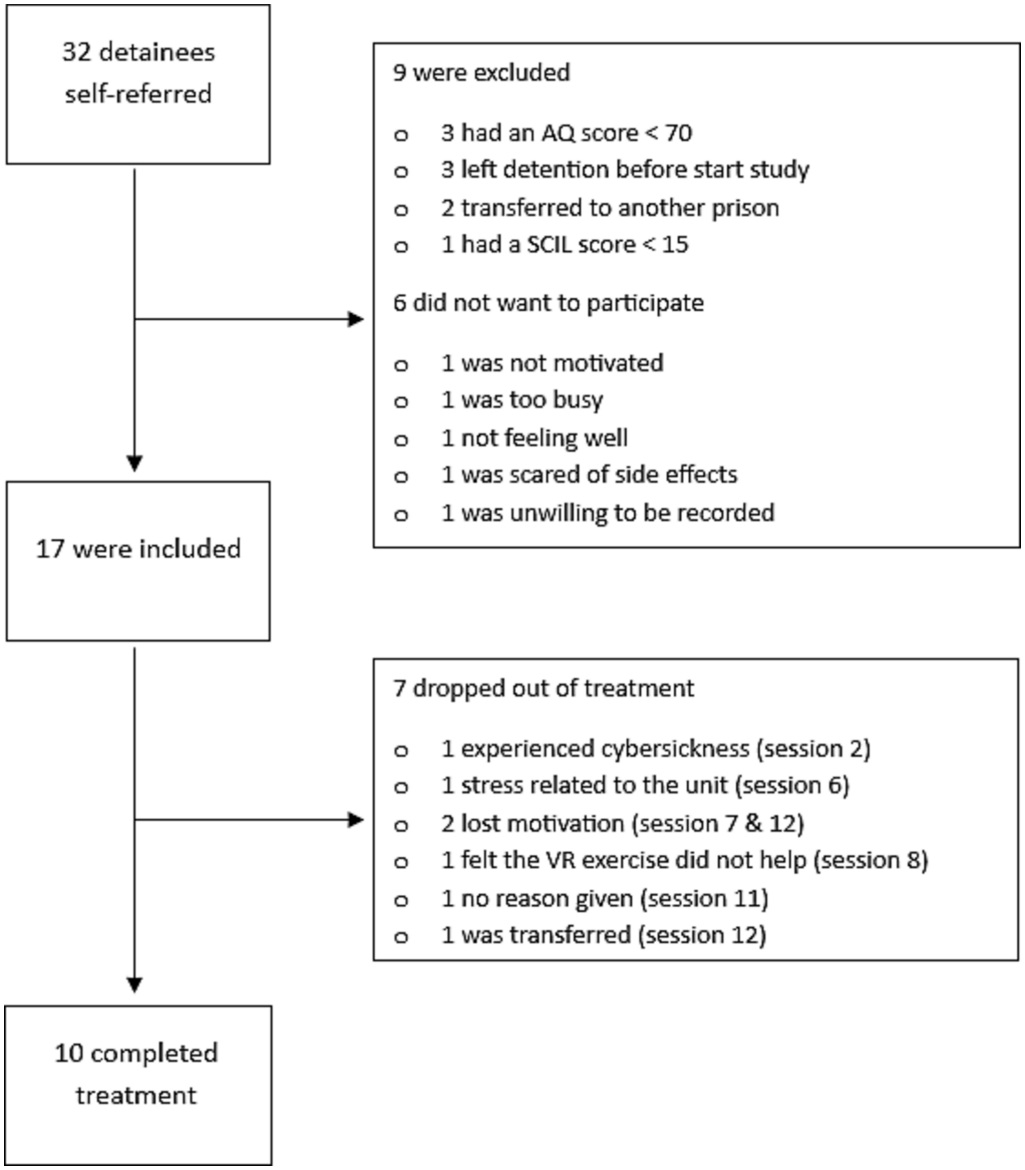
Flow chart of the pilot.

The study was completed between November 2019 and May 2021. In total, 32 detainees self-referred to the study. After the screening, 17 participants were included, see Figure 3. The baseline characteristics of the sample are shown in Table 1. Reasons for not participating after self-referral were: AQ score below 70 (*n* = 3), leaving detention before the start of the study (*n* = 3), transferal to another prison (*n* = 2), not motivated (*n* = 1), too busy (*n* = 1), not feeling well (*n* = 1), scared of potential side effects (*n* = 1), unwilling for physiological measurements to be recorded (*n* = 1), a SCIL score below 15 (*n* = 1), and no reason given (*n* = 1).

Of the 17 participants, 10 completed all 16 sessions. Reasons for dropout were cybersickness (*n* = 1 stopped after session 2), stress factors on the unit (*n* = 1 stopped after 6 sessions), loss of motivation (*n* = 2 stopped after session 7 and 12 respectively), feeling that the VR exercises did not help (*n* = 1 stopped after session 8), no reason given (*n* = 1 stopped after session 11), and transferal (*n* = 1 stopped after session 12).

Post-treatment measures were completed by all participants who finished the treatment, and by three of the participants who dropped out. Three of the 10 participants who completed VRAPT did not complete the follow-up assessment because of a lack of motivation, leaving detention and no specific reason; only one of the treatment dropouts completed the follow-up measure.

Feelings of presence in VR were slightly above the theoretical average of 3.5 on all subscales and were interpreted as acceptable (range 1–7): spatial presence was scored on average 4.0 (SD = 0.6), general presence 4.6 (SD = 2.1), involvement 4.2 (SD = 0.9) and experienced realism 3.6 (SD =1.1). After the first session, cybersickness symptoms were evaluated. Four participants reported that they experienced cybersickness, three of them described a feeling of dizziness, and one mentioned a feeling of ‘car-sickness’.

After each session the SRS was completed. Participants were predominantly positive about the VR exercises, sessions were rated with an average satisfaction score of 9.2 out of 10 (SD = 0.3; all data were included, also from participants who dropped out in a later session). Therapists indicated that overall, there was a good fit between the session content and the participants, see Table 2. A positive trend was noticeable, with almost only good ratings from session 7 onwards, during which the scenarios were more personalized due to interactive scenarios. Sessions with lower scores were session 3 and 6. In session 3 the theory of session 2 was repeated. In session 6 physiological measurements started, using the ‘aggression catwalk’ as exercise (which was also used in an earlier session). Therapists indicated that there were technical issues with the VR software or hardware in 22 sessions, and problems with the physiological measurements in six sessions.

**Table 2:** Match between session content and the participants aggression problems (therapist-rated).

	Session number													
	2	3	4	5	6	7	8	9	10	11	12	13	14	15
N	17	14	16	14	14	15	13	13	12	12	11	10	10	10
Good fit	82%	64%	88%	79%	64%	86%	100%	100%	83%	100%	100%	100%	100%	100%
Reasonable fit	0%	29%	12%	21%	21%	7%	0%	0%	17%	0%	0%	0%	0%	0%
No fit	18%	7%	0%	0%	14%	7%	0%	0%	0%	0%	0%	0%	0%	0%

In Table 3 the qualitative evaluation of the 17 participants and four therapists is presented. The three main topics included (1) what participants learned, (2) strengths of the treatment, and (3) points of improvement. Participants learned to respond more assertively and take more time to think about different reactions. They also learned to use more appropriate reactions in aggression-triggering situations. Participants gained more insight into the internal processes leading to aggressive behavior (such as triggers for aggression and estimating their level of tension), and insight into ascending aggression in others (e.g., in estimating aggression, emotions, and facial expressions of others). Participants as well as therapists were predominantly positive in their general opinion about the sessions. As a strength of the treatment, therapists mentioned that the scenarios played during the sessions fitted treatment goals well and were sufficiently challenging. Therapists mentioned that treatment provided different insights, e.g., in different forms of reacting, insight into their behavior and triggers. Points of improvement were also made. Therapists indicated that the theoretical part was too difficult to understand for some participants (such as the SIP model or the theory on emotions). Participants mentioned they wanted to practice more with the interactive scenarios and that these could be more personalized (such as more relatable scenarios, more challenging situations and customized to their circumstances). As for the physiological measurements, there were technical problems and results were found hard to interpret. As for the hardware and software, participants mentioned that the resolution and graphical realism could be improved, the walking speed could be faster and the ability to move more was missed (such as walking away by the participant).

**Table 3:** Qualitative evaluation of the treatment obtained from the workbooks by the therapists (*n* = 4) and participants (*n* = 17).

Topic	Explanation	Illustrative quotes
1. What participants learned		
1. Responding adequately & various ways of reacting	Participants reported that they learned during the treatment to respond assertively, take the time to think about a different reaction (different than aggression), deploy de-escalating behavior, and try to seek for a solution instead of discussing/win the argument.	‘That I also can react in a calm matter, I do not always need to react angry’ (*participant*) ‘That there are different ways of reacting in an assertive way. Walking away or taking your distance is not always sub-assertive’ *(participant)*
2. Coping strategies	Participants reported they learned about different coping strategies. In the role-plays, they frequently applied strategies such as staying calm, counting to ten, helping thoughts, and walking away.	‘Focus on your breath and walk away earlier from a situation’ *(participant)* ‘That I need to think about the consequences; what do I win by not reacting’ *(participant)*
3. Insight in self –triggers	Participants reported they gained more insight into their triggers for anger/aggression and what different reactions can evoke in others.	‘That there are various situations that trigger my anger, but it’s mostly about authority’ *(participant)* ‘I can react differently than I am used to; my reaction can evoke aggression with someone else; I was not aware of that’ *(participant)*
4. Insight in self – recognizing signs of aggression	Participants reported that they gained more insight into estimating aggression levels of themselves and more insight into their own tension levels.	‘It is important to pay attention to signals in your body, so tension does not raise’ *(participant)* ‘To come to a solution, you first need to calm down’ *(participant)*
5. Insight in others	Participants reported they gained more insight in estimating aggression levels, emotions, and facial expressions of others.	‘You need to listen and look carefully and estimate if someone really wants to hurt you’ *(participant)* ‘Someone’s physique does not automatically say something about his emotional state *(participant)*
2. Strengths of the treatment		
1. Scenarios triggered aggression	Therapists reported that the scenarios practiced in the treatment fitted well and were challenging enough for participants.	‘Participant did not bring in own scenarios, but said these were recognizable and fitted well’ *(therapist)*
2. Treatment provided insights in aggression	Therapists reported that sessions provided different forms of insight, as well as in aggression in general but also the theory provided per session.	‘It’s nice to see how he participates in the VR roleplays; he reacts fiercely on aggression. That’s the reason he gets in trouble often … he seems to understand well what we are doing in the treatment and why’ *(therapist)* ‘He seems to get more insight in where his aggression is stemming from’ *(therapist)*
3. Theory could be applied in VR	Therapists as well as participants were predominantly positive in their general opinion on how the sessions progressed and how the theory was applied in the VR role plays.	‘Participant managed to react in the way he had intended to’ *(therapist)* ‘Session went well, participant makes an effort to react in an assertive way and internalize it’ *(participant)*
3. Points of improvement		
1. Theory too difficult	Therapists reported that for some the SIP model was too hard to explain and apply, as well as the theory on emotions.	‘Difficult to explain facial expressions when someone really does not understand facial emotions’ *(therapist)* ‘Nice, but ill at ease. SIP model is hard to explain’ *(therapist)*
2. Practicing more with interactive scenarios	The interactive scenarios were seen as very useful by the participants, and this should be done more during the treatment and also in earlier sessions.	‘I wished to see more different reactions in the avatars, now they sometimes felt into repetition and that was less effective’ *(participant)*
3. More personalized scenarios	Participants reported that they would like to practice with more recognizable scenarios in the VR role plays.	‘It went well, but maybe more personalized things can be used’ *(participant)* ‘Add more recognizable situations’ *(participant)*
4. More challenging/ aggressive scenarios	Participants reported they would like to practice more with challenging and aggressive scenarios in the VR role plays.	‘There were no avatars who challenged me’ *(participant)’* ‘It could be more aggressive: say something about my family, be more personal: ‘come to my cell, we can work it out there or everybody will be informed about the address of your family’ *(participant)*
5. Personalizing sessions	Some participants mentioned that repetition was useful whereas others found it not useful. Some participants needed more sessions, some needed relaxation after the session.	‘Maybe a fun exercise to lose the tension after the session. I do not want to bring it to the unit’ *(participant)* ‘This session could be skipped, maybe it could be an optional session, so it could be rated per person if repetition is useful’ *(participant)*
6. Physiological measures	After session six it was asked therapists and participants what their experience with the physiological measurements was. Overall, it was concluded that the physiological measurements did not always work and results were hard to interpret.	‘Physiological measurements were difficult to understand (graphics) *(therapist)* ‘It’s difficult to pay attention to the heart rate and give feedback at the same time *(therapist)*. ‘Leave the ECG stickers out of the physiological measurements, they are annoying’ *(participant)*
7. Hardware/software	The hardware and software could be improved; it was reported that the resolution and realism could be higher, the walking speed was experienced as too low by some and that during interactive scenarios the ability to was missed.	‘Sometimes the VR world did not feel real’ *(participant)*

### 3.2 Intervention effects

Results of the self-report and observational measures over time are shown in Table 4. The mean scores of aggression, anger, provocation and emotion regulation decreased, with small to medium effect sizes. These improvements in mean levels were maintained at follow-up. Observational measurements showed a slight decrease in physical aggression at post-treatment, with a small effect size. This decrease was not maintained at follow-up. For verbal aggression, there was a small decrease for post-treatment as well as for follow-up, with a small effect size.

**Table 4:** Means, standard deviations, and test results of outcomes over time.

	Baseline		Post-treatment			Follow-up		
	*n*	Mean (SD)	*n*	Mean (SD)	Effect size	*n*	Mean (SD)	Effect size
Self-report measures								
Aggression (AQ) total score	17	98.6 (16.0)	14	90.7 (11.0)	0.37*	8	90.1 (16.9)	0.39^*^
Physical aggression	17	35.6 (6.7)	14	32.3 (6.9)	0.29	8	31.1 (8.0)	0.40
Verbal aggression	17	17.0 (2.7)	14	16.5 (2.7)	0.07	8	16.6 (4.3)	0.25
Anger	16	22.7 (4.9)	14	20.4 (4.3)	0.46	8	22.1 (4.6)	0.04
Hostility	17	23.1 (6.9)	14	21.6 (5.9)	0.09	8	20.3 (7.6)	0.56
Reactive & proactive aggression (RPQ) total score	17	23.1 (9.1)	14	21.5 (7.9)	0.29^*^	8	22.4 (11.7)	0.05
Reactive aggression	17	13.2 (4.3)	14	12.7 (4.1)	−0.01	8	12.8 (5.3)	0.14
Proactive aggression	17	9.9 (5.6)	14	8.9 (4.5)	0.21^*^	8	9.6 (6.5)	0.18^*^
Anger (NAS)	16	103.7 (14.8)	14	94.9 (15.5)	0.51	8	96.8 (13.2)	0.46
Cognitive	16	34.9 (4.7)	14	33.1 (5.0)	0.30	8	32.7 (3.0)	0.47
Arousal	16	34.7 (6.5)	14	30.5 (6.4)	0.66	8	32.4 (4.3)	0.30
Behavior	16	34.1 (5.6)	14	31.3 (5.7)	0.29	8	31.8 (6.9)	0.56
Provocation (PI)	15	60.7 (11.9)	14	55.3 (11.4)	0.31	8	53.4 (14.0)	0.48
Impulsiveness (BIS-11)	17	68.7 (13.9)	14	69.8 (14.8)	−0.46^*^	8	70.1 (17.1)	−0.11^*^
Emotion regulation (DERS)	16	94.3 (27.5)	13	85.9 (21.6)	0.28	7	69.1 (11.8)	0.58
Observational measures								
Physical aggression (SDAS)	17	0.35 (0.79)	17	0.20 (0.32)	0.24^*^	12	0.22 (0.47)	0.05*
Verbal aggression (SDAS)	17	4.95 (4.70)	17	4.07 (3.87)	0.19^*^	12	2.58 (2.56)	0.17^*^

Conventions effect size Hedges’ g correction for paired samples (0.20–0.49), medium (0.50–0.79), and large effect (≥0.80).

* Conventions effect size Wilcoxon signed-rank test effect size: small (0.10–0.29), medium (0.30–0.49), and large effect (≥0.50).

## 4 Discussion

We aimed to test the feasibility and acceptability of VRAPT, a VR treatment for aggression, in a prison-based population. Overall, therapists and participants were predominantly positive about the intervention and found it to be acceptable and feasible. The most commonly named strengths were the interactive roleplays which provided new insights in aggression of themselves and others. Points of improvement for the intervention were identified; the theoretical framework was too complex and sessions needed to be more customized to the needs of the participants. Results of the questionnaires and staff observations tentatively suggested improvements in aggression, anger, provocation and emotion regulation following the treatment.

### 4.1 Acceptability and feasibility

Overall, the recruitment went relatively quickly, with 32 self-referrals of detainees, even though no compensation was provided. Nonparticipation after self-referral occurred mainly because of external factors such as transfers, leaving detention or not meeting the criteria. These findings are in line with the first study on VRAPT, which also showed relatively easy recruitment (Klein Tuente et al., 2020).

Regarding treatment dropouts, two participants quit because of reasons unrelated to the intervention and four participants quit due to reasons either directly or possibly related to the intervention. In total 59% of the participants completed the treatment as intended. Although this number is relatively low, high dropout rates are common in this setting (Smeijers et al., 2018). Except for one participant, an inspection of the individual session satisfaction scores showed that even dropouts rated sessions between 7.5 and 10, suggesting that the dropouts due to motivation issues were unrelated to the intervention. Furthermore, it was noteworthy that drop-outs seemed to have experienced more trauma than completers of the treatment, they also seemed to be higher educated. However, these findings need to be further explored in future research with larger samples.

In general, participants and therapists were positive about VRAPT, as was shown by high session satisfaction scores by participants and good ratings by therapists on the fit between the session content and participants’ needs. Through the VR exercises, participants reported to have learned to respond more adequately to aggressive behavior, gained new insights into triggers and tension, and or gained insights into aggression and (facial) emotions of others.

Although the qualitative data showed that participants acquired several skills, it was also reported that not all scenarios were provocative enough and were sometimes hard to relate to. This indicates that therapists were sometimes too careful in acting out aggressive scenarios, reflecting a different point of view between the participants and therapists when it comes to aggressive behavior. What was seen as highly aggressive by therapists can be mildly aggressive for participants. Furthermore, there needs to be more room for discussing personal situations where aggression occurs so that more relevant scenes can be roleplayed.

Therapists needed some time to adjust to the software and hardware, but over time they got more experienced and familiar with it. Extensive training and practice are needed when first starting to use VR. Some technical problems were mentioned, but these could be fixed in a relatively short time with the support of the helpdesk or by restarting the software. Therapists also reported some issues with physiological measurements. They found it difficult to play interactive scenarios and simultaneously understand and give feedback on heart rate and skin response, resulting in less usage than planned in the protocol.

Although feelings of realism and presence in VR were moderate, this did not seem to have immediate implications for the treatment. Concerning realism, no feedback was given by participants on improving the VR environments. There are indications that other factors are of more importance in how VR is perceived, such as the involvement of emotion or arousal in VR (Ling et al., 2014; Diemer et al., 2015). In accordance with this, several studies in the last two decades have used VR software that was less realistic but nonetheless effective (Freeman et al., 2017; Pot-Kolder et al., 2018).

### 4.2 Treatment effects

While demonstrating the efficacy of VRAPT was not an aim of this pilot, nearly all effects were in the expected direction showing improvements between baseline and post-treatment (except for impulsiveness). Self-reported aggression improved between baseline and posttreatment with medium effect size, and the effect was maintained at follow-up. Further, outcomes on anger, provocation, and emotion regulation decreased with small to medium effect sizes. It is important to notice that questionnaires concerning aggression focused on trait aggression, which is also affected by other factors such as childhood trauma (Sarchiapone et al., 2009), and may influence effects. Small improvements in staff-observed aggression were found, especially in verbal aggression. SDAS observations only focus on aggressive behavior, and newly learned positive (coping) behavior is thus not scored.

Whereas scores of most measures stabilized between posttreatment and follow-up, emotion regulation improved further in the period after the treatment. This finding seems to fit well with the treatment targets, and converges with the qualitative findings which reported that participants learned new coping skills and gained insights in estimating signs of their aggression which is a precursor for being able to regulate such emotions. Maladaptive emotion regulation is common in offenders (Roberton et al., 2014). A recent study showed that emotion regulation moderates the effect between anger and aggression in aggressive offenders (Xie et al., 2023), further stressing the importance of intervening on emotion regulation. This could also indicate that longer follow-up periods are relevant for aggression outcomes, as in response to improved emotion regulation, aggression might decrease over time as well. As such, it would be interesting to explore in future research whether emotion regulation mediates or moderates treatment effects on aggression.

The results of the current study are in line with the earlier VRAPT study of Klein Tuente et al. (2020), as mean scores on aggression, anger and provocation (as measured with the AQ and NAS-PI) changed in similarly in both studies. It is noticeable that mean baseline scores of aggression on both the AQ and RPQ were overall higher in this study than the study of Klein Tuente and colleagues, which may result in detecting small changes in aggression easier in this prisoner population.

### 4.3 Adjustments to the treatment protocol

Based on this pilot, several adjustments were made to the treatment protocol. First, the theory explained during the treatment was minimized and simplified as criticism by the therapists indicated the theory was hard to understand and apply for participants. Instead of explaining SIP steps in each session in-depth, the model is now used as an underpinning for the therapists and is explained explicitly to participants during session 1 only. As a more practical replacement, elements from cognitive behavioral therapy (CBT) are used, providing insights in the relation between thoughts, feelings, behavior, and the consequences of behavior in a specific situation. Also, theory was linked directly to the different exercises to make it more practical and understandable.

Second, homework assignments were added to both increase treatment efficacy and enable creating more personalized and challenging VR scenarios. Participants were initially encouraged to apply what they learned in each session in daily life. However, this turned out to be difficult for most of them. To give more guidance, participants have to complete forms on the think-feel-act-consequence (the CBT-related exercise) as a homework exercise about aggressive or stressful events that week (Beck, 2011). The forms will also be used as input for creating personal scenarios. In the pilot, participants found it hard to come up with concrete examples for personal scenarios, and the new homework assignment can help with this.

Third, to enable more personalized sessions and practice more in VR during the treatment, we replaced two sessions (sessions 4 and 9) with two sessions in which the therapist and participant can freely choose what to practice. In this way, the treatment can be tailored more to the individual and specific assignments with which the participant has difficulty can be practiced.

Finally, physiological measurements were removed from the treatment protocol as the data was too difficult to monitor and interpret for therapists during roleplays (which resulted in minimal usage). Furthermore, the ECG was experienced as uncomfortable by participants. However, as it was relevant to discuss experienced tension, an ‘anger thermometer’ was added to session 5 and 8 which is a common tool for discussing tension (Rose et al., 2008).

### 4.4 Limitations

This study had several limitations. First, participation was based on self-referral, which may have led to selection bias. Participants may have been more motivated for (innovative) treatment than the average prison population. However, treatment in Dutch prisons is always on a voluntary basis. No further documentation was kept on the reasons why participants wanted to participate, which is a limitation. Also, only males were included in this study, so we do not know whether the findings would be the same or different in females.

Data were missing for some questionnaires, and questions concerning feedback on the protocol and study were open-ended questions in the workbook of the therapist that were not always fully completed. Also, it is unknown which other forms of care participants received during the study period.

Furthermore, participants completed the qualitative questions in the workbooks in the presence of the therapist, which may have caused a positive bias as therapists were not blinded and participants may have been less critical and may have given more socially desirable answers due to the presence of the therapist. To minimize positive bias it was emphasized that therapists shared no content-related information with any other parties and that participating or dropping out could not influence (positive or negative) ongoing trajectories in detention in any way, to clarify for participants that there were no further gains from participating. Also, during the study period, VRAPT therapists only had contact with participants for VRAPT and not for any other reason.

Finally, only about half of the participants completed the follow-up measurement. We checked whether participants who did not complete measurements differed in the main outcome from completers at the start of the study. This was not the case, baseline aggression total scores revealed that both participants who completed the follow-up measure (M = 97.9, SD = 15.9) and who did not complete the measure (M = 99.2, SD = 16.9) had similar levels of aggression at the start of the study. Thus, this does not seem to have caused a bias in aggression outcomes.

### 4.5 Conclusion

Our findings indicate that VRAPT is an acceptable and feasible intervention for both detainees and therapists to train multiple skills for reducing aggressive behavior. Furthermore, preliminary positive findings on aggression, anger, and emotion regulation suggest that this treatment has potential in a prison-based population. Implementing VRAPT in a larger-scale RCT requires several adjustments, such as simplifying the theoretical framework and roleplaying with more personalized scenarios. Based on our findings we have adjusted the treatment protocol to a new version specifically for detainees called Virtual Reality-Treatment for Aggression Control (VR-TrAC). Our next step will be to test VR-TrAC in an RCT.

## Data availability statement

The raw data supporting the conclusions of this article will be made available by the authors upon request, without undue reservation.

## Ethics statement

The studies involving humans were approved by Medical Ethical review board UMC Groningen PO Box 300,019,700 RB GRONINGEN. The studies were conducted in accordance with the local legislation and institutional requirements. The participants provided their written informed consent to participate in this study.

## Author contributions

WV, EM, and SK designed the study. KW collected the data. KW and CG wrote the first draft of the manuscript. KW and CG carried out the analysis of the results. All authors contributed to the article and approved the submitted version.
